# Innovative Nanomaterial Properties and Applications in Chemistry, Physics, Medicine, or Environment

**DOI:** 10.3390/nano14020145

**Published:** 2024-01-09

**Authors:** Thomas Dippong

**Affiliations:** Faculty of Science, Technical University of Cluj-Napoca, 76 Victoriei Street, 430122 Baia Mare, Romania; dippong.thomas@yahoo.ro

## Introduction

Developing innovative nanomaterials unlocks new opportunities in physics, chemistry, medicine, and environmental protection [1,2,3,4,5,6]. Controlling and designing nanomaterials with precise properties (size, morphology, porosity, chemical, mechanical, photocatalytic, and magnetic properties) became a highly explored research topic. Particular emphasis involved interdisciplinary research, which merged expertise from physics, chemistry, materials science, and biomedicine with targeting for optimized nanomaterial performance [7,8,9,10,11,12,13,14,15].

Metallic-based (chromates, ferrites, bismuthates, aluminates, etc.) and carbon-based (carbon nanotubes, graphene, graphene oxides, etc.) nanomaterials are the most explored [3,4,5,6,7,8,9,10]. Nanoparticles’ properties make them appropriate applicants for technical applications in biosensors, humidity sensors, photocatalysis, magnets, drug delivery, magnetic refrigeration, magnetic liquids, photoluminescence, microwave absorbents, ceramic pigments, gas sensing, corrosion protection, water decontamination, antimicrobial agents, biomedicine, and catalysis [10,11,12,13,14,15,16,17,18,19,20,21,22,23,24,25]. Adjusting the nanoparticles’ shapes, sizes, and properties can be performed via the synthesis route, by modifying parameters such as the pH and concentration of reactants, dopants, or thermal treatment temperature and time [20,21,22,23,24,25,26,27,28,29].

This Special Issue, “Innovative Nanomaterial Properties and Applications in Chemistry, Physics, Medicine, or Environment”, focused on (i) the synthesis and characterisation of nanoparticles, nanotubes, nanowires, or nanofibers; (ii) nanostructures (graphene, zeolites, membrane, etc.), coatings, and thin films; (iii) the correlation between chemical composition, morphology, surface, and magnetic properties of nanostructured materials; (iv) the thermal behaviour of ceramic pigments with applications in glazes; (v) nanomaterials for photocatalysis and electrocatalysis; (vi) nanomaterials for water purification; (vii) nanomaterials for adsorption of organic and inorganic pollutants; and (viii) nanomaterials for biosensing and biomedical applications. This Special Issue includes 12 original research papers, and highlights the development of synthesis and characterization of nanomaterials and nanostructures of different natures with various applications in chemistry, physics, medicine, biology, and the environment.

Dippong et al. [30] described the influence on the structure, morphology, and magnetic properties of Co_0.4_Zn_0.4_Ni_0.2_Fe_2_O_4_ nanoparticles embedded in a SiO_2_ matrix. The production of low crystalline Co–Zn–Ni ferrite at 500 °C and highly crystalline Co–Zn–Ni ferrite was accompanied by traces of crystalline Fe_2_SiO_4_ at 800 °C. At 1200 °C, the size of spherical particles increased with the Co_0.4_Zn_0.4_Ni_0.2_Fe_2_O_4_ ferrite content (36–120 nm) [30]. The magnetic properties were enhanced with an increased ferrite content and annealing temperature. The non-embedded Co_0.4_Zn_0.4_Ni_0.2_Fe_2_O_4_ was ferromagnetic with a high M_S_, while the SiO_2_ matrix was diamagnetic with a minor ferromagnetic portion. Non-embedded Co_0.4_Zn_0.4_Ni_0.2_Fe_2_O_4_ displayed a single magnetic phase, while the Co_0.4_Zn_0.4_Ni_0.2_Fe_2_O_4_ embedded in the SiO_2_ matrix showed two magnetic phases. The particle size and key magnetic parameters were diminished by embedding the ferrite into the SiO_2_ matrix [30].

The influence of doping with various monovalent (Ag^+^, Na^+^), divalent (Ca^2+^, Cd^2+^), and trivalent (La^3+^) metallic ions and annealing temperatures (500, 800, 1200 °C) on the physico-chemical properties of MnFe_2_O_4_@SiO_2_ ceramic nanocomposites produced by the sol–gel method was discussed by Dippong et al. [31]. Low crystalline MnFe_2_O_4_ at low annealing temperatures and well-crystalline MnFe_2_O_4_ at high annealing temperatures were noted. At 1200 °C, in the case of MnFe_2_O_4_ doped with divalent and trivalent metallic ions, three crystalline phases belonging to the SiO_2_ matrix were also noted. The structural parameters determined by X-ray diffraction, namely the spherical particles size, the thickness of coating layer, and the powder surface area, as well as the magnetic parameters (*M_s_*, *M_r_*, *K*, *H_c_*), depended on the doping metallic ion and annealing temperature [31]. By doping, the *Ms* and *K* decreased for samples annealed at 800 °C, but increased for samples annealed at 1200 °C, while the *M_R_* and *H_c_* decreased by doping at 800 and 1200 °C [31].

The impact of La^3+^ doping and annealing on the structure, morphology, and photocatalytic properties of Ni ferrite was also reported by Dippong et al. [32]. The XRD validated the formation of a single-phase cubic spinel structure at all annealing temperatures. The particle and crystallite sizes decreased from 37 to 26 nm by substituting Fe^3+^ with La^3+^ ions, while the lattice constants increased with an increase in La^3+^ content and annealing temperature, owing to the different La^3+^ and Fe^3+^ ionic radii [32]. The specific surface area varied with the La^3+^ content, and decreased with the increase in the treatment temperature, possibly due to the larger particle size and higher crystallinity at higher temperatures [32]. The excellent sonophotocatalytic performance was reported for NiLa_0.3_Fe_1.7_O_4_/SiO_2_, most likely due to the La^3+^ content inserted in the band gap of Ni ferrite [32].

Atanasov et al. [33] reported the magnetic properties and magnetocaloric effect of polycrystalline and nano-manganites Pr_0.65_Sr_(0.35−x)_Ca_x_MnO_3_, obtained by a solid-state reaction and the sol–gel method. Rietveld’s refinement confirmed the presence of a single phase with orthorhombic (Pbnm) symmetry, and lower cell volume and Mn–O bond length at a higher Ca substitution [33]. The bulk samples’ resistivity was related to the grain boundary conditions and ferromagnetic/paramagnetic conversion [33]. Curie temperature values decreased from 295 K to 201 K, with an increasing Ca substitution. Due to the magnetocaloric property and the tuned Curie temperature, the obtained bulk polycrystalline compounds could be capable candidates for magnetic refrigeration [33].

The simple fabrication of active CeO_2_@ZnO nanoheterojunction photocatalysts with various Zn:Ce ratios by pyrolysis of Ce/Zn-MOFs precursors was reported by Ai et al. [34]. By increasing the Zn:Ce ratio from 0 to 1, pure CeO_2_, ZnO, and CeO_2_@ZnO nanocomposites were obtained [34]. With the Zn:Ce ratio increasing, the CeO_2_@ZnO nanocomposites progressively converted from nanospheres into nanoparticles of about 10 nm [34]. The optical band gaps widened with the increase in Zn:Ce ratio, due to the quantum size effects and heterojunction interface [34].

Jaramillo-Fierro and León [35] reported the impact of doping TiO_2_ nanoparticles with lanthanides (La, Ce, and Eu). Moreover, the photodegradation of cyanide, the impact of reactive oxygen species on the photocatalytic procedure below simulated light, and the reuse of these nanoparticles in five successive cycles were tested [35]. The highest percentage of cyanide removal was reported for La/TiO_2_ (98%), Ce/TiO_2_ (92%), Eu/TiO_2_ (90%), and TiO_2_ (88%). According to the obtained results, the La, Ce, and Eu dopants enhanced the adsorption and photocatalytic properties of TiO_2_ semiconductors, as well as their ability to remove cyanide from aqueous synthetic solutions [35].

The study by Segura-Sanchis et al. [36] focused on scanning photocurrent microscopy (SEM) in single-crystal multidimensional hybrid lead bromide perovskites. Their composition played an important role in their optoelectronic properties, mainly the effect on the photogenerated transport charges along the microcrystals [36]. Noteworthy changes among multidimensional perovskite crystals regarding the photocarrier decay length values and spatial dynamics across the crystal were also noted [36]. The photocurrent maps indicated that the effective decay length cuts near the border, indicating the importance of border recombination centres in monocrystalline samples [36]. The multidimensional 2D–3D perovskites showed a single exponential fitting model, while the 3D perovskites confirmed a fast and slow charge carrier migration dynamic inside the crystal [36].

Kim et al. [37] reported the 3D plasmonic architecture of Ag nanoparticles functionalized with carbon nanowalls, and their use in increasing the hotspot density [37]. The evaluation of the substrate using Rhodamine 6G in concentrations of 10^−6^ to 10^−10^ M for a 4 min exposure established a related increase in Raman signal intensity with the concentration [37]. The large specific surface area and graphene domains of carbon nanowalls provide dense hotspots and high charge mobility, contributing to the electromagnetic and chemical mechanisms of surface-enhanced Raman spectroscopy [37]. The pioneering SERS concept embraces a high potential for bioanalysis and chemical analysis [37].

The recent breakthroughs in using quantum dots, i.e., semiconducting tiny nanocrystals of 2–10 nm with tunable optoelectronic properties, for cancer imaging and drug delivery purposes, were reviewed by Hamidu et al. [38]. This research emphasized semiconducting quantum dots’ synthesis methods and properties, such as slight tunable size, great surface-to-volume ratio, steady photoluminescence, and excellent biocompatibility [38]. The conjugation or binding of molecules to the quantum dots’ surfaces were also discussed. The properties of these nanoparticles prevail over the limitations of traditional cancer management approaches, and may allow for early detection and treatment [38].

The study by Khalaj-Hedayati et al. [39] focused on identifying and in silico characterisation of a preserved peptide on an influenza hemagglutinin protein. The computational framework was used to evaluate the efficacy of an antigen produced over current bioinformatics tools [39]. Epitope mapping approached five top-ranked B-cell and twelve T-cell epitopes, and the consistent Human Leukocyte Antigen alleles to T-cell epitopes exposed a high population coverage [39]. The theoretical physicochemical properties of HA2_88–107_ peptides guaranteed the thermostability and hydrophilicity, while the obtained results indicated the obtaining of a capable antigen for an influenza vaccine strategy [39].

Fritz et al. [40] examined the distribution of the particle size and shape of the in vivo bioproduced particles from aqueous Au^3+^ and Eu^3+^ solutions using the cyanobacterium *Anabaena* sp. An incubation time of 51 h doubled the number of Au particles, and the spherical diameter shrank to 8.4–7.2 nm [40]. *Anabaena* sp. could rapidly bioform small-sized nanoparticles displaying a high tendency towards sphere-shaped nanoparticles [40]. Accordingly, *Anabaena* sp. is a suitable applicant for the cost-effective and non-toxic production of a specific nanoparticle size and shape by varying the growth time [40].

The adsorption outcome of CH_4_ molecules on monolayer PbSe was investigated, considering the first-principle calculations by Zhou et Mao [41]. The impact of the adsorption of CH_4_ molecules on monolayer PbSe, and the Se and Pb vacancies of monolayer PbSe, indicated that the CH_4_ molecules display an excellent physical adsorption effect on monolayer PbSe with/without vacancy defects [41]. The variations in the band gap were more sensitive to strain for the monolayer PbSe with Se vacancy. In this regard, the adsorption capacity of the CH_4_ molecules on the strained system decreased sharply, suggesting that strain can successfully regulate the electronic structure of CH_4_ molecules adsorbed on a 2D PbSe nanomaterial [41]. Moreover, the Se vacancy and P-PbSe displayed diverse reactions on the CH_4_ molecule adsorption when using the same strain [41].

Considering that the variety of innovative compounds, and that the rapid development of investigating tools in the multidisciplinary research of metal-based nanomaterials is continuously progressing, this Special Issue will underwrite the interest of research in this area by providing a broad and updated scenario. These published research papers will indicate a new line for future studies to produce significant advances related to materials science and engineering.

Concluding, as the Editor of this Special Issue, I would like to thank all of the authors and reviewers who contributed to this Special Issue with their innovative ideas and constructive comments. I am also grateful for the consistent support from the *Nanomaterials* Editorial Office. Indeed, this Special Issue will provide an accessible platform to comprehend the synthesis and characterisation of innovative nanomaterials and nanostructures and their crucial roles in various applications.
